# Liver safety of two nucleoside analogs plus efavirenz, nevirapine or a ritonavir-boosted protease inhibitor in HIV/HCV-coinfected drug-naïve patients

**DOI:** 10.1186/1758-2652-13-S4-P91

**Published:** 2010-11-08

**Authors:** J Macías, J Mallolas, LF López-Cortés, JA Cartón, P Domingo, S Moreno, JA Iribarren, K Neukam, A Rodrigo, MJ Jiménez-Expósito, JA Pineda

**Affiliations:** 1 Hospital Universitario de Valme, Unidad Clínica de Enfermedades Infecciosas, Seville, Spain; 2 Hospital Clinic, Servicio de Enfermedades Infecciosas, Barcelona, Spain; 3 Hospital Universotario Virgen del Rocio, Servicio de Enfermedades Infecciosas, Seville, Spain; 4 Hospital Central de Asturias, Unidad de Enfermedades Infecciosas, Oviedo, Spain; 5 Hospital de la Santa Creu i Sant Pau, Unidad de Enfermedades Infecciosas, Barcelona, Spain; 6 Hospital Universitario Ramón y Cajal, Unidad de Enfermedades Infecciosas, Madrid, Spain; 7 Hospital de Donostia, Unidad de Enfermedades Infecciosas, San Sebastian, Spain; 8 Bristol-Myers Squibb, Madrid, Spain

## Purpose of the study

A wide range of hepatotoxicity rates associated with antiretroviral drugs (ARV) has been reported in different studies. Reasons for this include diverse populations, with different prevalence of viral hepatitis coinfection, exposure to diverse ARV and variable criteria to define liver toxicity across studies. The liver safety of regimens including efavirenz (EFV), nevirapine (NVP) or boosted-protease inhibitors (PI/r) has not been compared in a single large cohort of HIV/HCV-coinfected patients with a uniform definition of hepatotoxicity. Because of this, we evaluated the incidence and risk factors for grade 3 or 4 ALT or AST elevations (TE) and grade 4 total bilirubin elevations (TBE) among ARV-naïve HIV/HCV-coinfected patients with an initial regimen including two nucleoside analogs (NRTIs) plus EFV, NVP or a PI/r.

## Methods

Retrospective multicenter cohort (January 2000-June 2006) of 745 HIV/HCV-coinfected patients starting their first ARV treatment consisting of two NRTIs plus either EFV, NVP or PI/r. Patients were included if they were exposed to ARV for one week or more and had evaluations within the first three months of follow-up. Definition of grade 3 or 4 TE: 1) Normal baseline ALT or AST levels: ALT or AST elevations ≥5 times above the upper limit of normal (ULN); 2) Baseline ALT or AST levels above the ULN: ALT or AST elevations ≥3.5 times the baseline values Definition of grade 4 TBE: Increases of total bilirubin ≥5 mg/dl.

## Summary of results

323 (43%) patients received EFV, 126 (17%) NVP and 296 (40%) PI/r. Grade 3 or 4 TE were observed in 19 (5.9%) individuals on EFV, 14 (11%) on NVP and 31 (10.5%) on PI/r (global comparison, p=0.068; EFV vs. NVP, p=0.056; EFV vs. PI/r, p=0.036; NVP vs. PI/r, p=0.38). Grade 4 TBE were identified in 7 (2.2%) patients on EFV, 1 (0.8%) on NVP and 11 (3.7%) on PI/r (p=0.19). Severe TBE was detected in 5 (12%) and 15 (2%) patients with and without cirrhosis, respectively (p=0.003). Discontinuations due to hepatotoxicity were: 13 (4%) patients on EFV, 16 (13%) on NVP and 17 (6%) on PI/r (global comparison, p=0.003; EFV vs. NVP, p=0.001; EFV vs. PI/r, p=0.320; NVP vs. PI/r, p=0.015).

## Conclusions

Liver tolerability of regimens including EFV, NVP or PI/r is generally good in ARV-naïve HIV/HCV-coinfected patients. Severe TE are less commonly seen with EFV than with NVP or PI/r. Discontinuations due to hepatotoxicity were less frequent for patients receiving EFV than for those treated with NVP.

